# Perspective: Designing T-Cell Engagers With Better Therapeutic Windows

**DOI:** 10.3389/fonc.2020.00446

**Published:** 2020-04-15

**Authors:** Omid Vafa, Nathan D. Trinklein

**Affiliations:** Teneobio, Inc., Newark, CA, United States

**Keywords:** T-cell engager, bispecific, CD3 redirection, cancer, therapeutic window, cytokine release

## Abstract

This perspective highlights the history and challenges of developing CD3-based bispecific T-cell engagers (TCEs) as cancer therapeutics as well as considerations and potential strategies for designing the next generation TCE molecules. The goal of this article is to raise awareness of natural T-cell biology and how to best harness the tumor cell killing capacity of cytotoxic T-cells with TCEs. In light of 30 years of concerted efforts to advance TCEs in early clinical development, many of the first-generation bispecific antibodies have exhibited lackluster safety, efficacy, and manufacturability profiles. As of January 2020, blinatumomab remains the only approved TCE. Many of the current set-backs in early clinical trials implicate the high-affinity CD3 binding domains employed and the respective bispecific platforms as potential culprits. The underlying conviction of the authors is that by taking corrective measures, TCEs can transform cancer therapy. Through openness, transparency, and much needed feedback from ongoing clinical studies, the field can continuously improve the design and effectiveness of next generation T-cell redirecting therapeutics.

## Introduction: Heeding Nature'S Design

When considering the design of TCEs, it is important to appreciate the characteristics of immune-recognition and the biology of T-cells which we aim to redirect. Antibody-producing B-cells and T-cells are the effector cells that carry out the adaptive immune response and specifically recognize foreign proteins on infected or cancerous cells. T-cells recognize foreign peptides on infected or mutated cells through T-cell receptors (TCR) that bind foreign peptide-human leukocyte antigen complexes (pHLA) at low affinities ranging from 1 to 100 uM (1–3). Low affinity binding of the T-cell receptor to its cognate antigen is an important feature of the T-cell immune response. Consequently, the T-cell response is driven by avidity-based antigen recognition through multiple low-affinity TCRs (3–5). The TCR is a multi-protein complex that includes the CD3 subunits that translate cell surface antigen binding into an intracellular phosphorylation signaling cascade. These phosphorylation events culminate in the activation of transcription factors such as NFAT and NFkB that lead to increased expression of cytokines and effector proteins such as granzymes and perforin (5, 6). The intensity of signaling through TCR complexes ultimately determines T-cell fate, including cytolytic activity, proliferation, exhaustion, and apoptosis. Complementing pHLA:TCR complex signaling, both costimulatory and coinhibitory T-cell receptor pathways modulate the balance of controlled T-cell activation. It was through the understanding of these pathways that a number of therapeutics (anti-CTLA4, anti-PD-1, and anti-PDL1) were developed to modulate T-cell activation against cancers expressing neoantigens and overcome the immune-suppressive microenvironment of tumors (7, 8).

A key observation relating to TCR signaling was highlighted by two different groups nearly two decades ago. These studies showed that induction of T-cell cytolytic activity does not require the formation of a stable and mature immunological synapse (9, 10). Importantly, Faroudi et al. noted that the activation threshold for target cell lysis was >1,000-fold more sensitive than the activation threshold for cytokine release, and that this difference was primarily due to differences in antigen concentration on the cell surface of target cells and the number of pHLA:TCR complexes formed. Together, these published studies established the dual threshold model of T-cell activation. The implications of this model along with the low affinity of natural TCR binding events are important considerations for determining the design parameters of T-cell engaging bispecific antibody therapeutics.

## A Brief History of CD3- Based T-Cell Engagers

A TCE is a protein that simultaneously binds through a target antigen on a tumor cell and CD3 on a T-cell to form a TCR-independent artificial immune synapse and circumvent HLA restriction. The earliest efforts using CD3 binding antibodies for T-cell activation date back the mid-1980's when studies of heteroaggregates of anti-CD3 (T3, from OKT3 hybridoma) showed anti-cancer cytotoxicity (11). The first published description of a bispecific TCE was of a rat isotype hybrid generated by Clark and Waldmann (12), who demonstrated targeted killing of TH-1 cells. Shortly after in 1990, a chemically conjugated TCE was created and used to demonstrate the first clinical proof-of-concept for treating malignant glioma in Japan (13). After a lull in clinical development of bispecifics due in large part to manufacturing complications, the field witnessed the clinical success of catamuxamab, an anti-EPCAMxCD3 mouse-rat hybrid bispecific administered intraperitoneally for malignant ascites (Fresenius Biotech, Germany, EMA approval in 2009, voluntarily withdrawn in 2017). Soon after, Micromet Inc. (Germany, USA) initiated trials for blinatumamab, a mouse anti-CD19xCD3 dual single chain variable fragment (scfv)-based bispecific, administered intravenously for acute lymphoblastic leukemia (ALL) (Amgen, CA, FDA approval in 2014).

While these early studies showed promising clinical efficacy, they were also hampered by severe dose-limiting toxicities primarily manifesting as cytokine release syndrome (CRS). This resulted in prohibitively narrow therapeutic windows and was due in large part to the anti-CD3 binding domains that were used. A comprehensive review of the literature shows that many early TCE drug developers relied on three primary mouse-derived anti-CD3 antibodies: OKT3, SP34, and UCHT1 (14–17). These original CD3 antibodies bind with a relatively high affinity in the single to low double-digit nM range. As described earlier, this is roughly 1,000-fold higher affinity than a natural pHLA:TCR interaction and likely has profoundly different effects on the activation of T-cells compared to natural signaling through the TCR.

After considering the limitations of first-generation TCEs in the context of the natural function of T-cells, we must re-think how we approach and engineer the next generation of bispecific T-cell engagers. Invoking the systems thinking motto of “optimizing subcomponents of a system does not necessarily optimize the overall system,” it is worth reassessing our approach to multi-specific antibody development and the interdependencies of their structural and functional components. In a recent instructive review, Ellerman (18) provided a comprehensive perspective on the variables that can impact T-cell engagement. They include the antibody format, epitopes bound on CD3, membrane proximity of the epitope bound on the tumor antigen, target binding affinity, half-life, etc. (18). Mandikian (19) further highlighted importance of CD3 affinity of TCEs and their impact on tissue distribution. High affinity CD3-binders of HER- targeting TCEs were shown to distribute preferentially to secondary lymphatic tissues, reducing systemic exposure. In contrast, a high affinity tumor antigen binding domain was also suboptimal if rapidly internalized, with low residence time on the cell surface (19). In addition, when including an Fc to increase half-life of TCEs, a critical consideration is the elimination of Fc receptor interactions. Significant off-target toxicities (20, 21) and CRS that can arise from inadvertent cross-linking of standard Fc-containing bispecifics through adjacent Fc receptor-expressing cells (22), and active Fcs can potentially negatively impact *in vivo* efficacy (23). Arguably, when considering the aforementioned variables impacting TCE safety and efficacy, the failure of many early TCE therapeutic molecules may be a consequence of combining binding domains that were individually optimized but were not optimized to work together.

**Figure 1:**
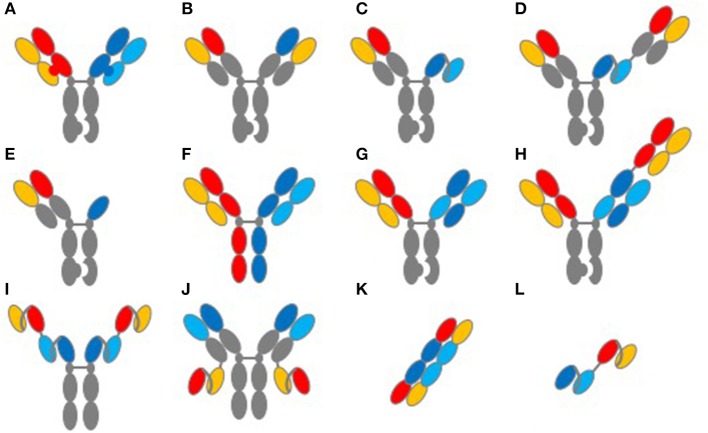
Common structures of TCE proteins. This figure illustrates common molecular formats used to create TCE proteins. **(A)** knob-into-hole format for Fc and light-chain heterodimerization. **(B)** knob-into-hole format using a common light chain. **(C)** knob-into-hole triple-chain format, HC:LC Fab paired with scFv (Xencor) and **(D)** the 2+1 format including a second Fab (Xencor). **(E)** knob-into-hole triple-chain format, HC:LC Fab paired with heavy-chain only binding domain (Teneobio). **(F)** Fab arm exchange, DuoBody® (Genmab). **(G)** knob-into-hole Cross-MAb 1+1 format (Roche) and **(H)** knob into hole CrossMAb 2+1 format (Roche). **(I)** tetravalent scfv Fc fusion and **(J)** tetravalent HC:LC and scfv fusion (NV Cheung, MSKCC). **(K)** TandAb diabody (Affimed). **(L)** tandem scFv, first generation BiTE®format (Amgen).

When considering the interdependencies of TCE structure and function, it is important to highlight the antibody format used and its impact on developability. A summary of commonly used formats for TCEs is shown in Figure 1. In addition to the biological complexities of initiating an artificial immune synapse, one of the key challenges with TCEs has been in the generation of fully human bispecific formats that are biophysically soluble, stable and manufacturable at large scale. Advances in antibody engineering since the 1990's have enabled an exponential increase in the number of formats and scaffolds that can be used in assembling bispecifics [Figure 1 and reviewed in detail in (22, 24, 25)]. In these endeavors, the use of human sequences and the elimination of biophysical liabilities such as the amino acid residues that undergo post-translational modifications remain essential to producing therapeutic proteins. Specifically, TCE protein aggregates can have serious safety implications, given their potential to prematurely activate T-cells in the absence of target engagement. Enabling long-term stability of robust and non-immunogenic platforms will be key to the clinical advance of platforms to commercialization.

A challenge related to the biological mechanism of action of early TCEs derives from past patterns of thinking. Early TCE efforts were biased toward developing molecules with the most potent cytotoxic activity based on *in vitro* cell-based assays without anticipating the biological consequences of high potency on cytokine release and T-cell exhaustion or depletion in the patient. These observations and safety concerns were summarized at a recent FDA-sponsored workshop focused on CD3 TCE safety assessment (26). Blinatumomab's small size and short half-life requires step-wise dosing (initial 9 μg/d followed by 28 μg/d by continuous infusion), which enables a steady Cmax to avoid neurotoxicity and CRS at higher concentrations (27). The second generation of TCEs include Fcs or other domains conferring half-life extension. Based on publicly reported adverse events and clinical holds in the last few years, the prospect of extending half-life with a high potency TCE could exacerbate serious adverse events associated with neurotoxicity and CRS. To address the complications associated with high potency anti-CD3 antibodies, companies like Xencor (Pasadena, CA) and Macrogenics (Gaithersburg, MD) mutated the SP34 anti-CD3 antibody to humanize and reduce binding affinity in efforts that demonstrated reduced cytokine release *in vitro* and *in vivo* (28, 29). Nevertheless, it remains to be determined whether reduced-affinity anti-CD3 TCEs will improve therapeutic window since the original SP34 anti-CD3 binding domain remains suboptimal in the clinic. Preventative measures for CRS have relied on pre- or co-medication with corticosteroids as well as anti-IL6R (tociluzimab) to ameliorate grade 3 and 4 adverse events. Whether such treatments also compromise the efficacy of TCEs is a matter of current debate.

## The Next Generation OF T-Cell Engagers

**Figure 2:**
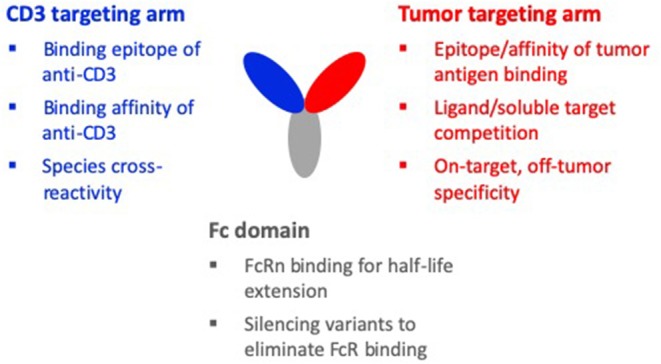
Design considerations of a TCE molecule. The three binding domains of a typical TCE molecule are the CD3 targeting arm, the tumor targeting arm, and the Fc domain. When designing the CD3 targeting arm, important considerations are: the binding epitope, the binding affinity, and species cross-reactivity. For the tumor targeting arm, important considerations are: the epitope and affinity of tumor antigen binding, competition with soluble target or a ligand of the target, and on-target, off-tumor specificity. Considerations for the Fc domain are: maintaining FcRn binding for half-life and silencing variants to eliminate FcR binding, complement activity, and non-specific CD3 clustering and T-cell activation. For the holistic design of a TCE molecule, these design considerations must be made in the context of the interdependencies of the different domains.

Due to the limitations of the first and second generation TCEs that relied on re-purposing mouse-derived CD3 antibodies such as OKT3, SP34, and UCHT1, more recent efforts have focused on discovering new CD3 binders and adopting the principles of holistic design. Figure 2 summarizes the design considerations for the CD3 binding domain in the context of the other binding domains of a TCE molecule. With these considerations in mind, the goal of new discovery efforts is to identify CD3 binding antibodies that are fully human and bind new epitopes on the CD3 complex with a range of affinities. Most importantly, these new CD3 antibodies are meant to be “fit-for-purpose,” designed and functionally screened specifically for optimal behavior in TCE bispecific antibodies. Toward this goal, we at Teneobio (Newark, CA) have discovered numerous novel human anti-CD3 binding domains through sequence-based discovery of fixed light chain transgenic rats (30).

Based on the previous work of Faroudi et al. (9), our goal was to identify leads which preferentially trigger the cytolytic activity of T-cells and avoid the production and secretion of large quantities of pro-inflammatory cytokines. Characteristic of one of the CDR families (F2) was that its members uniquely bound a conformational epitope that recognizes the CD3δε heterodimer preferentially over CD3γε and over a wide range of affinities from low to high nanomolar (30). Importantly, in the context of human IgG heterodimeric bispecific antibodies, F2 family members retained full efficacy against cancer target cells while demonstrating low levels of cytokine release (30). Consistent with our results, recent studies by Zuch de Zafra et al. (31) and Li et al. (32) also demonstrated that T-cell mediated cytotoxicity can be decoupled from cytokine release when using TCEs. Li et al. further showed that initial release of TNF from T-cells was the primary culprit driving CRS by triggering downstream proinflammatory cytokine release from monocytes. Moreover, Teneobio's F2 family members can preferentially activate CD8+ cells over regulatory T-cells (unpublished data). This differential activation is noteworthy and therapeutically relevant, given that Duell et al. (33) showed that blinatumomab (based on the anti-OKT3 scaffold) can activate Tregs and thereby inhibit T-cell proliferation and killing. Finally, unlike the first generation anti-CD3 TCEs, F2 family-based TCEs do not upregulate T-cell inhibitory receptors such as PD1 and CTLA4, which are hallmarks of T-cell exhaustion and/or anergy (unpublished data). This unique attribute of the F2 family binders is likely due to signaling intensity driven by CD3 affinity and the distinct binding epitope on CD3δε. Importantly, TCE developers using platforms based on OKT3 should take heed of the fact the OKT3 is apoptotic in the presence of IL-2 (34) and that clinical studies involving humanized OKT3 (teplizumab, hOKT3g1) to treat type I diabetes demonstrate that teplizumab induces T-cell exhaustion as well as apoptosis of CD8+ T-cells (35). These observations have obvious clinical relevance and pose potential liabilities when selecting OKT3-based binders for TCE platforms.

An additional consideration when designing a TCE with a better therapeutic window is whether decoupling cytotoxicity from cytokine release can impact maximal efficacy, especially for solid tumors. In theory, completely eliminating IFNγ production could minimize its anti-tumoral effects and dampen downstream immune stimulation from HLA class I upregulation (36). On the other hand, IFNγ can also upregulate PD-L1, posing unwanted tumoricidal resistance, necessitating PD-L1 blockade (37). The ideal level of cytokine production and how the pleiotropic effects of cytokines impact the efficacy of next generation of TCEs is the subject of current debate and will require further investigation in preclinical models and human patients.

Beyond identifying TCE-optimized CD3 binding domains, a number of companies are exploring alternative approaches to designing therapeutics which can reduce cytokine release and improve safety. Biotech companies like CytomX (South San Francisco, CA), Maverick Therapeutics (Brisbane, CA), and Amunix (South San Francisco, CA) have introduced proteolytic sites in their therapeutic molecules whereby local tumor cell proteases can cleave and conditionally activate the respective highly potent TCEs at the site of the tumor, potentially minimizing systemic toxicities. These various formats are currently in preclinical stages of development and undergoing IND-enabling studies. The success of these platforms will undoubtedly rely on their stability post-manufacturing and the retention of the conditionally activated bispecific at the tumor site with minimal diffusion that may impact on-target off-tumor cytotoxicity.

## Future Opportunities and Challenges

Early clinical results and the new improvements to TCE design has spurred the discovery and clinical advance of 66 bispecific TCEs that are now in Phase I and Phase II studies as of January 7, 2020. Current clinical studies of TCEs span both hematological (39 trials) and solid tumors (34 trials), with over a hundred additional programs in preclinical development (personal communication with Paulina Szymanska, Beacon Target Therapies). Not surprisingly, most pharma and biotech companies are pursuing hematological cancers by targeting lymphocyte restricted tumor-associated antigens such as CD19, CD20, BCMA, CD33, and CD123. Importantly, as disclosed in the most recent American Society of Hematology (ASH) abstracts in December of 2019, a number of novel TCEs targeting BCMA and CD20 are showing favorable and complete responses in myeloma and lymphoma patients, respectively (38–42).

While the early clinical results with TCEs in hematological cancers are showing impressive efficacy, solid tumors represent a patient population that is 10-times larger with an even greater unmet medical need. One of the major goals in the field of TCE is effectively addressing solid tumors. To this end, multiple companies in pharma and biotech are pursuing TCEs targeting common, over-expressed solid tumor antigens such as HER2, PSMA, EPCAM, and CEA. Others are pursuing pHLA neoantigens as targets using T-cell receptor (TCR) protein scaffolds (e.g., Immunocore, UK) and TCR mimics comprising antibody scaffolds that recognize HLA-peptide complexes (Eureka Therapeutics, CA, Gritstone Oncology, CA). However, it is unlikely that TCEs can simply be applied to solid tumors in the same way they are used in hematological cancers. Solid tumor cancers are fundamentally different diseases compared to hematological cancers (43). Unlike many of the B-cell targets whose expression is limited to the B cell lineage, the aforementioned solid tumor antigen targets are not exclusively restricted to tissues of origin associated with specific cancers. Therefore, TCEs targeting solid tumor-associated antigens must address safety concerns related to “on-target, off-tumor” activity in healthy tissues (26, 44). One way this is being addressed is with a bivalent CEA-targeting TCE (2+1 format) that preferentially targets high expressing CEA on solid tumors while avoiding low expressing primary cells (45). Another example is a HER2-targeting TCE that uses multi-valent avidity-based HER2 binding that biases activity to tumor cells with the highest antigen density (46). With this multi-valent antigen binding design, the low level of HER2 expression on cardiac cells and other healthy tissue is insufficient to induce T-cell engagement and activation in mouse models of HER2-positive breast cancer.

In addition to tumor specificity, other significant challenges in treating solid tumors with TCEs are overcoming the immunosuppressive tumor microenvironment (TME) and the physical barriers to cytotoxic T-cell trafficking and tumor penetration defined as the stroma (47). Solid tumors recruit immunosuppressive cells such as myeloid derived suppressor cells (MDSCs), tumor-associated macrophages (TAMs), and regulatory T-cells (Tregs), all of which inhibit the activity of cytotoxic T-cells. Therefore, the most effective use of TCEs in solid tumors will likely require using TCEs combined with agents such as checkpoint inhibitors and stroma disrupters that help to overcome the immunosuppressive TME and render an immune excluded or immune desert “cold” tumor into an inflamed “hot” one (48). In addition to checkpoint blockade, antibody agonists to co-stimulatory targets such as CD28 and CD137 as well as immune-activating cytokines such as IL-2 and IL-15 can promote the expansion of peripheral T-cells and lower the threshold for T-cell activation and are being investigated as ways to overcome immunosuppression in solid tumors. In this context, it is essential that TCEs have a favorable safety profile and broad therapeutic window when used in combination to address solid tumors.

Combination treatments that break the stroma barrier, comprising basement membrane, fibroblasts and the extracellular matrix, could enable T-cell penetration. These may involve the use of antibody drug conjugates or alpha-emitters to stroma cells, targeting fibroblast activation protein alpha (FAP-alpha), or the FGF and TGF-ß pathways (47, 49). Other approaches involve targeting stellate cells, hyaluronan, and secreted extracellular matrix (ECM) associated proteins (50, 51). A number of preclinical proof of concept studies show the feasibility of some of these aforementioned approaches [reviewed in (47)], which will be ripe for early clinical experimentation in combination with TCEs, pending favorable outcomes of current ongoing clinical trials (e.g., see clinical trials.gov for Phase I and II studies of sibrotuzumab (NCT02198274), Fresolimumab (NCT02581787), defactinib (NCT03287271), and AZD4547 (NCT01791985). Ultimately, we anticipate that TCEs with improved therapeutic windows may afford favorable synergies in solid tumor treatment with checkpoint inhibitors, stroma disrupters, targeted co-stimulatory agents or cytokines, and other modulators of the solid tumor microenvironment.

## Concluding Remarks

The old alchemical phrase, “*In sterquiliniis invenitur*” translates to “in filth it will be found.” Implicit in this message is that what you need most can be found in the mess where you least wish to look. This phrase can be applied to the clinical development of TCE therapeutics where biological complexity and clinical failures are ever-present challenges. Our industry could improve the transparency with which we share the details of failures in both TCE preclinical and clinical development. Not knowing the basis for such failures can delay faster and informed development of better TCEs. Openness and learning from both preclinical and clinical outcomes will enable continuous improvement in building better molecules for meaningful therapeutic benefits to patients.

## Author Contributions

All authors listed have made a substantial, direct and intellectual contribution to the work, and approved it for publication.

### Conflict of Interest

OV and NT are employees of Teneobio, Inc. with equity interests.
